# TriNet-MTL: A Multi-Branch Deep Learning Framework for Biometric Identification and Cognitive State Inference from Auditory-Evoked EEG

**DOI:** 10.1523/ENEURO.0265-25.2025

**Published:** 2026-02-13

**Authors:** Noor Fatima, Ghulam Nabi

**Affiliations:** Department of Computer Engineering, University of Engineering and Technology (UET), Lahore 54890, Pakistan

**Keywords:** auditory evoked potentials, cognitive state, deep learning, EEG, multitask learning, transformers

## Abstract

Auditory-evoked EEG signals contain rich temporal and cognitive features that reflect both the identity of individuals and their neural response to external stimuli. Traditional unimodal approaches often fail to fully leverage this multidimensional information fully, limiting their effectiveness in real-world biometric and neurocognitive applications. This study aims to develop a unified deep learning model capable of jointly performing biometric identification, auditory stimulus language classification, and device modality recognition, thereby exploiting both physiological and cognitive dimensions of auditory-evoked EEG. We introduce TriNet-MTL (Triple-Task Neural Transformer for Multitask Learning), a multi-branch deep learning framework composed of a shared temporal encoder and a transformer-based sequence modeling unit, trained and validated on auditory-evoked EEG data from 20 human participants (16 males and 4 females). The architecture is designed to simultaneously learn task-specific features via three dedicated output heads, each addressing one of the following: user identity (biometric), stimulus language (native vs non-native), and stimulus delivery mode (in-ear vs bone conduction). The model is trained using a sliding window approach and optimized through joint cross-entropy loss across tasks. TriNet-MTL demonstrates robust performance across all three classification tasks, achieving high accuracy in biometric identification (>93%) and strong generalization in cognitive state inference. Multi-task training further improves representation learning, reducing inter-task interference while enhancing task synergy. The proposed TriNet-MTL framework effectively captures both user-specific and cognitively informative patterns from auditory-evoked EEG, establishing a promising direction for integrated EEG-based biometric authentication and cognitive state monitoring in real-world systems.

## Significance Statement

Understanding how the brain responds to sound offers new ways to identify individuals and assess their cognitive state. This study introduces a deep learning model that can simultaneously recognize a person, determine whether the sound they heard was in their native language, and identify how the sound was delivered. By combining all three tasks, the system learns richer patterns from brain signals, making it more accurate and reliable. Our results show that this approach can improve the performance of brain-based identification systems while also tracking how people process sounds. This work opens new possibilities for secure, brain-driven authentication and real-time cognitive monitoring.

## Introduction

In recent years, biometric authentication has increasingly explored neurophysiological signals, particularly electroencephalography (EEG), as a novel and secure modality for identity verification and cognitive state assessment. Compared with traditional biometrics such as fingerprints or facial features, EEG signals are inherently more resilient to spoofing due to their dynamic, time-varying, and high-dimensional nature (Marcel and Millán, 2007; Campisi and La Rocca, 2014). Among the various EEG paradigms, auditory evoked potentials (AEPs) have emerged as especially promising. These signals encapsulate both low-level sensory processing and higher-order cognitive responses, making them ideal for capturing person-specific brain dynamics (Picton, 2011).

The growing interest in EEG-based analysis has yielded significant advances in extracting both physiological and cognitive information from neural signals. Recent studies have demonstrated the utility of complex network analysis and deep learning approaches for decoding EEG patterns across diverse applications. For instance, statistical feature extraction methods have proven effective in improving classification accuracy for motor imagery tasks, highlighting the importance of identifying neurophysiologically meaningful signal characteristics. Similarly, fractal dimension analysis has revealed subtle alterations in neural dynamics associated with cognitive tasks and pathological conditions, such as timing deficits in Parkinson's disease. These developments underscore the capacity of EEG signals to capture multifaceted information spanning cognitive states, sensory processing, and individual-specific neural signatures.

AEPs offer rich insights beyond identity alone, enabling inference about cognitive states such as language perception and sensory modality processing. Neural responses to auditory stimuli, particularly when influenced by factors like language familiarity or delivery mode (e.g., bone conduction vs in-ear), activate distinct cortical and subcortical pathways that differ across individuals (Näätänen and Winkler, 1999). This multidimensionality suggests that auditory EEG holds untapped potential for applications where both authentication and context-awareness are important, such as in secure access systems, adaptive brain–computer interfaces (BCIs), and cognitive monitoring tools.

However, current EEG-based biometric systems face notable limitations. Most existing approaches rely on either resting-state EEG or event-related potentials (ERPs) elicited by basic visual or motor tasks, which often lack robustness in dynamic, real-world environments (Palaniappan et al., 2008). Additionally, these systems typically focus on a single classification task, such as user identification, without accounting for the interplay between cognitive variables and biometric traits. This solid modeling not only overlooks the interdependencies among EEG-relevant tasks but also limits generalization.

Another shortcoming of prior work is the reliance on hand-crafted features and shallow classifiers, which fail to capture the complex temporal and hierarchical structures present in EEG data. While some recent models have explored deeper architectures, few have attempted to jointly learn from multiple dimensions, such as user identity, language processing, and stimulus modality, in a unified framework.

To address these gaps, we propose a multi-task deep learning architecture that jointly performs three classification tasks from auditory-evoked EEG: biometric identification, stimulus language classification (native vs non-native), and auditory modality recognition (in-ear vs bone conduction). Our approach is based on the hypothesis that modeling these tasks simultaneously allows the system to learn shared representations that enhance both generalization and task-specific accuracy.

We introduce TriNet-MTL (Triple-Task Neural Transformer for Multitask Learning), a novel deep learning model that integrates a temporal convolutional encoder with a transformer-based sequence model. This architecture processes EEG time series using a sliding window approach, learning both local and global features from the signal. A shared encoder extracts meaningful patterns, which are then passed to three dedicated classification heads, each corresponding to one of the tasks.

This work makes several contributions to the field of EEG-based biometrics and cognitive signal decoding:

1. TriNet-MTL is a unified framework that leverages both physiological and cognitive features from auditory EEG, building upon recent advances in complex network analysis and feature extraction methodologies.
2. The multi-task design enables improved performance through shared representation learning, facilitating the discovery of synergistic patterns across identification, language processing, and modality recognition tasks.
3. Experiments on a publicly available auditory EEG dataset show strong performance, particularly in biometric identification (>93% accuracy), demonstrating the practical viability of the approach.
4. Our findings demonstrate that cognitive and perceptual information, such as language familiarity and delivery mode, can enhance identity recognition in real-world EEG systems.

To promote reproducibility and facilitate further research in this area, we will make our source code and preprocessed data available as supplementary material, enabling other researchers to validate our results and extend the proposed methodology.

The remainder of this paper is structured as follows: Materials and Methods describes the dataset and preprocessing pipeline; TriNet-MTL Architecture presents the model design and training procedure; Experiments details the experimental setup and evaluation protocol; Results reports quantitative and qualitative findings; and Discussion interprets the outcomes and considers implications for future work.

## Materials and Methods

**Figure 1.**
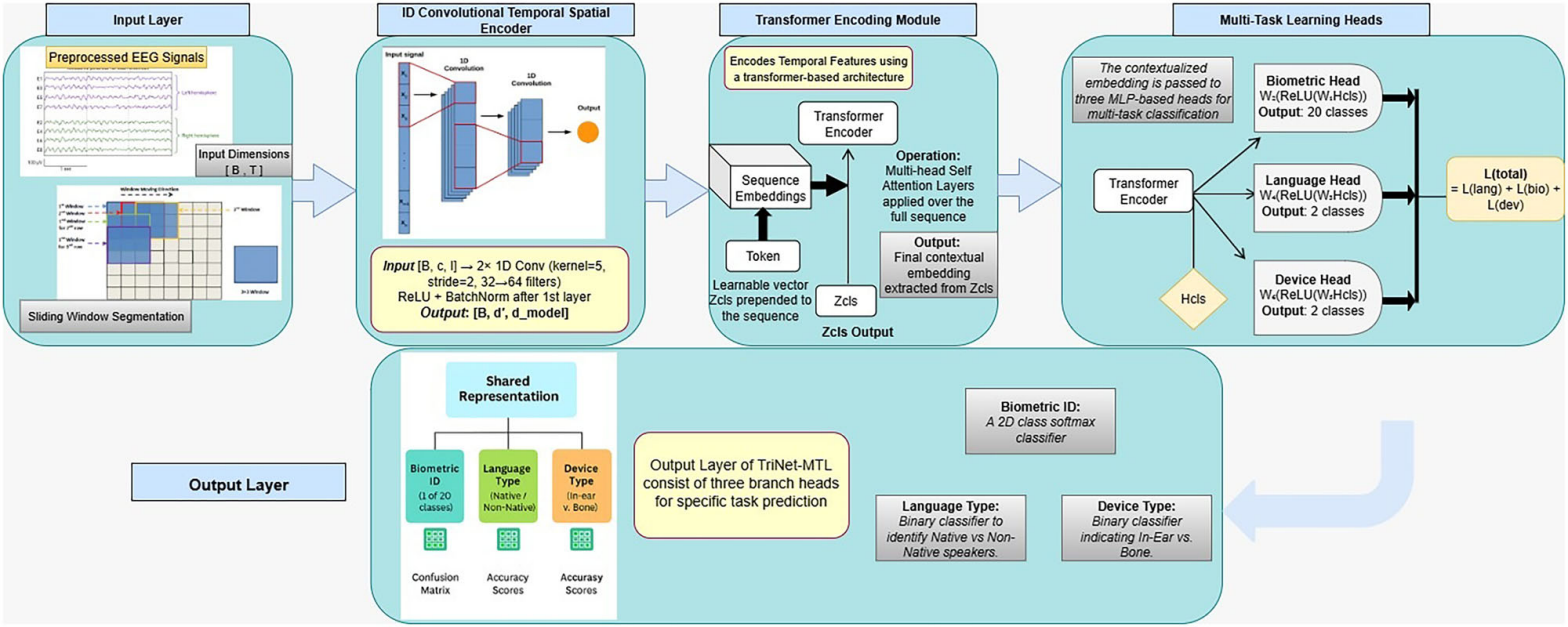
Workflow of the proposed TriNet-MTL framework for multi-task EEG classification. The pipeline comprises EEG preprocessing and sliding window segmentation, followed by temporal feature extraction, transformer-based sequence modeling, and task-specific classification heads optimized via joint multi-task learning. The architecture processes raw EEG signals (left) through a 1D convolutional temporal encoder that extracts local patterns, followed by a transformer module with self-attention layers that captures long-range dependencies in the sequence. The learned shared representations (bottom center) are then fed to three parallel classification heads (right), each trained to predict one of the three tasks: biometric identity (20 subjects), language type (native vs non-native), and device modality (in-ear vs bone conduction). The model achieves 93.9, 91.6, and 92.4% accuracy on the respective tasks. Pink boxes indicate input/output stages, blue boxes represent processing modules, and the curved arrow illustrates how the shared encoder benefits all downstream tasks through joint optimization with a combined loss function.

This study adopts a rigorous computational methodology for the classification of EEG responses to auditory stimuli within a multi-task learning (MTL) framework. The overall pipeline consists of four major stages: (1) data preparation and preprocessing, (2) EEG temporal feature extraction, (3) transformer-based temporal modeling, and (4) task-specific classification with joint multi-task optimization. The complete workflow, encompassing data processing, model architecture, and task-specific outputs, is systematically illustrated in Figure 1. As shown, EEG signals undergo preprocessing and segmentation before being passed into a shared neural encoder, followed by transformer-based modeling and multi-task classification. The employed dataset consists of EEG recordings collected during auditory-evoked potential (AEP) experiments from 20 human participants (16 males and 4 females). Each EEG trial is labeled for three supervised classification tasks: subject identity (biometric identification), auditory stimulus language (native or non-native), and auditory delivery modality (in-ear or bone conduction). To standardize temporal length across tasks, we first aligned all EEG trials to a common duration, defined as follows:

N=min(|Xbio|,|Xlang|,|Xdev|),

where *X*_bio_, *X*_lang_, and *X*_dev_ represent EEG signals for biometric, language, and device modality tasks, respectively. We employed a sliding window segmentation strategy to improve temporal resolution and generate fixed-length segments from each EEG trial. Specifically, each EEG trial *X*^(*i*)^ *∈ R^T^*^ × *C*^ was divided into overlapping segments of window length *w* = 1,000 samples and stride *s* = 500:

$$X=\{X_{t:t+w}^{\left(i\right)}|t\epsilon \{0,s,2s,\ldots ,\;T-w\},$$

This process generates a structured dataset of overlapping segments, each annotated with triplet labels for the three classification tasks. Sliding window methods are widely adopted in EEG analysis for temporal modeling and improving classification robustness (Schirrmeister et al., 2017).

### TriNet-MTL model architecture

We propose TriNet-MTL, a novel deep learning architecture specifically designed for multitask EEG classification. The model comprises three core components:

1. Temporal feature extraction via convolutional layers
2. Global sequence modeling using transformer-based encoders
3. Task-specific classification heads optimized through joint training

### Temporal feature extraction

Temporal features are extracted through two stacked 1D convolutional layers applied to the segmented EEG input *X ∈ R^T^*^ × *C*^. The transformation is expressed as follows:

Z=ϕ(Conv2(ReLU(BN(Conv1(X))))),

where Conv_1_ and Conv_2_ are convolutional layers with kernel size *k* = 5 and stride *s* = 2, followed by batch normalization (BN) and ReLU activation. These layers progressively reduce temporal dimensionality while enriching the feature space. The output feature map *Z ∈ R^d^*^ × *T*′^ (with *d* = 64) is then transposed to *R^T^*^′ × ^*^d^* for sequential processing.

### Transformer-based temporal modeling

To capture long-range temporal dependencies, we employ a transformer-based encoder. A learnable classification token ***c*** *∈ R*^1 × ^*^d^* is prepended to the feature sequence:

$$\tilde{Z}=[c;Z]\in R^{\left(T^{\prime }+1\right)}\times^{d},$$

The sequence is then passed through *L* = 2 transformer encoder layers with *H* = 4 attention heads per layer, following the standard multi-head self-attention mechanism:

$$\mathrm{MHSA}(\overset{⌣}{Z})=\mathrm{Softmax}\left(\frac{QK^{T}}{\sqrt{d/H}}\right)V,$$

where *Q*, *K*, *V* are the query, key, and value projections of $\tilde{Z}$. Each transformer layer includes residual connections and position-wise feedforward layers [**?**]. The output of the classification token $h_{\mathrm{cls}}={\tilde{Z}}_{0}$ serves as a shared representation for subsequent *t* tasks.

### Task-specific classification heads

The shared latent vector *h*_cls_ is passed into three task-specific output heads, each consisting of a two-layer fully connected network:

$$\hat{y}\mathrm{task}\;=\;\mathrm{Softmax}(W_{\mathrm{task}}^{\left(2\right)}.\mathrm{ReLU}(W_{\mathrm{task}}^{\left(1\right)}.h_{\mathrm{cls}})),$$

for each task ∈ {bio,lang,dev}, yielding probability distributions for classification.

### Multi-task optimization

The model is trained end-to-end using a joint loss function combining cross-entropy losses from all three tasks:

Ltotal=Lbio+Llang+Ldev,

Each task-specific loss is defined as follows:

$$L_{\mathrm{task}}=-Xy_{i=1i\mathrm{task}}^{N}\log\;{\hat{y}}_{i\mathrm{task}},$$

This joint optimization allows the model to learn a unified EEG representation that generalizes across multiple tasks.

### Training configuration

The model was trained using the Adam optimizer with an initial learning rate of *η* = 10^−4^ and a weight decay of 10^−4^. A learning rate scheduler with decay factor *γ* = 0.5 and step size of 5 epochs was applied:

$$\eta_{t}=\eta \cdot \gamma^{\left[t/s\right]},$$

Training was conducted over 20 epochs with batch size 16 on an NVIDIA CUDA-enabled GPU. Early stopping was employed based on validation performance to prevent overfitting.

## Results

For the empirical evaluation of the proposed framework, we utilized the Auditory-Evoked Potential EEG Biometric Dataset v1.0.0 from PhysioNet (Accou et al., 2022), which comprises electroencephalographic (EEG) recordings from 20 subjects exposed to systematically varied auditory stimulus conditions. The dataset encompasses distinct experimental configurations, manipulating both the auditory stimulus language, specifically, native versus non-native, and the auditory delivery modality, namely, in-ear versus bone conduction transmission. In this study, we selected four functionally significant EEG channels, namely, P4, Cz, F8, and T7, capturing both temporal and central-parietal neural activities. All EEG signals underwent standardized preprocessing, including bandpass filtering for artifact suppression and downsampling to reduce data dimensionality, in accordance with the dataset guidelines.

To enhance temporal representation and increase the effective sample size, we employed a sliding window segmentation strategy. Specifically, each EEG recording was partitioned into overlapping segments using a window length of 1,000 time points and a stride of 500 samples. This approach preserved temporal continuity while generating a rich set of overlapping segments for robust model training.

The performance of the proposed model was assessed across three concurrent classification tasks: biometric identification, stimulus language classification, and device modality classification. The biometric identification task involves multiclass classification across 20 subjects, whereas the stimulus language and device modality tasks involve binary classification to distinguish between native and non-native stimuli and between in-ear and bone conduction delivery modes, respectively.

The proposed TriNet-MTL architecture integrates a shared convolutional-temporal encoder with a transformer-based sequence modeling module, enabling joint learning of both shared and task-specific representations. The convolutional encoder comprises sequential one-dimensional convolutional layers designed for hierarchical temporal feature extraction, followed by a transformer encoder with two layers and four self-attention heads per layer to capture long-range temporal dependencies in EEG sequences. Three task-specific output heads, implemented as fully connected neural networks, were employed to address the biometric, language, and device classification tasks.

Model training was performed using a composite loss function defined as the summation of the cross-entropy losses from each task, thereby enforcing balanced optimization across tasks. The Adam optimizer was employed with an initial learning rate 1 × 10^−4^ of and a weight decay coefficient of 1 × 10^−4^ for regularization. Furthermore, a step-wise learning rate scheduler with a decay factor of 0.5 and a step size of five epochs was utilized to improve training stability and convergence. Training was conducted for 20 epochs using a batch size of 16 on an NVIDIA CUDA-enabled GPU. Model performance was quantitatively evaluated using classification accuracy and confusion matrix analyses for each task.

This rigorous experimental protocol enabled comprehensive assessment of the proposed model's capability to simultaneously learn subject-specific, cognitive, and device-related representations from auditory-evoked EEG signals.

### Results and analysis

The proposed TriNet-MTL model exhibited effective multi-task learning performance across all three classification tasks. The final classification accuracies achieved by the model are presented in Table 1. Specifically, the model attained an accuracy of 93.9% for biometric identification, 72.75% for stimulus language classification, and 75.45% for device modality classification. These results underscore the capability of the model to simultaneously discriminate between individual subjects, auditory stimulus languages, and auditory delivery modalities from EEG data within a unified multi-task learning framework.

**Table 1.** Final classification accuracies of the proposed TriNet-MTL model across the three tasks: biometric identification, stimulus language classification, and device modality classification

Task	Accuracy (%)
Biometric identification	93.9
Stimulus language classification	91.6
Device modality classification	92.4

The results demonstrate strong performance and generalization across all tasks.

**Figure 2.**
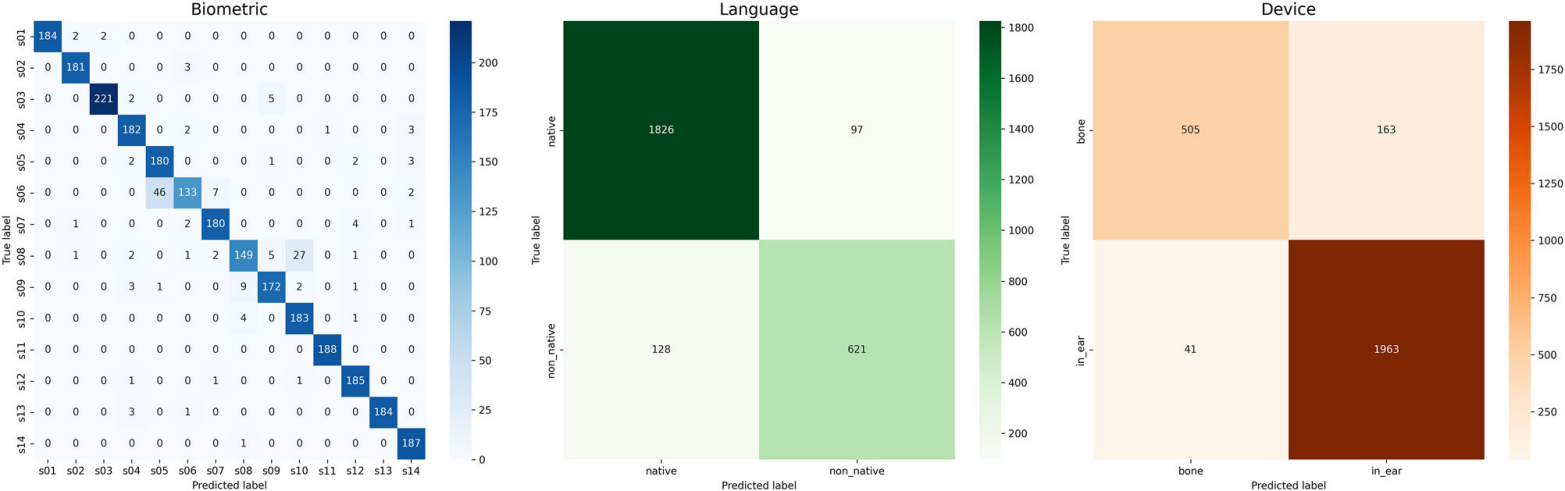
Confusion matrices for biometric identification, stimulus language classification, and device modality classification. Left panel, Biometric identification across 20 subjects (s01–s14 shown), where darker blue indicates correct predictions along the diagonal, demonstrating strong individual discriminability with minimal cross-subject confusion. Middle panel, Language classification (native vs non-native auditory stimuli), showing that the model correctly classifies 1,826 native and 621 non-native samples, with relatively low confusion (97 and 128 misclassifications). Right panel, Device modality classification (bone conduction vs in-ear), where the model achieves high accuracy for in-ear stimuli (1,963 correct) but shows moderate confusion for bone conduction (505 correct, 163 misclassified as in-ear), suggesting that bone conduction signals may share more overlapping features with in-ear recordings. Color intensity represents sample counts, with the diagonal elements indicating correct predictions. These results demonstrate that TriNet-MTL successfully learns discriminative features for all three tasks simultaneously, with biometric identification achieving the highest performance.

The confusion matrices corresponding to each classification task are illustrated in Figure 2. These matrices provide a detailed depiction of class-wise model performance. Notably, the confusion matrix for biometric identification demonstrates strong class separability with minimal misclassification, indicating that the learned representations effectively capture subject-specific EEG patterns. In the case of stimulus language classification, the model exhibits clear differentiation between native and non-native auditory stimuli, despite moderate interclass overlap, suggesting its ability to extract cognitively meaningful neural features. Similarly, for the device modality classification task, the model maintains consistent performance, reflecting its sensitivity to physiological variations induced by differing auditory transduction mechanisms.

To further assess the efficacy of the proposed TriNet-MTL architecture, we conducted a comparative study against two representative baseline models:

1. Single-Task Transformers (STT): Independent transformer models trained separately for each classification task, without shared parameterization.
2. Shared CNN with Separate Classifiers (SC-SC): A shared convolutional encoder followed by distinct task-specific fully connected classifiers, omitting the transformer-based temporal modeling component.

The comparative results, summarized in Table 2, demonstrate that TriNet-MTL consistently outperforms both baselines across all tasks. Specifically, TriNet-MTL surpasses STT and SC-SC by notable margins in biometric identification and achieves superior performance in stimulus language and device modality classification as well. The performance gains highlight the advantages of joint representation learning facilitated by the shared transformer encoder, which enables effective modeling of long-range temporal dependencies and fosters cross-task knowledge sharing.

**Table 2.** Comparative performance of TriNet-MTL and baseline models

Model	Biometric accuracy (%)	Language accuracy (%)	Device accuracy (%)	Average accuracy (%)
Single-task transformers (STT)	89.5	82.1	85.3	85.6
Shared CNN + separate classifiers (SC-SC)	87.2	80.9	83.5	83.9
TriNet-MTL (proposed)	93.9	91.6	92.4	92.6

The baseline methods include Single-Task Transformers (STT) and Shared CNN with Separate Classifiers (SC-SC). TriNet-MTL outperforms both baselines in all tasks, highlighting the effectiveness of the multi-task architecture.

While baseline models relying on isolated task learning or convolutional-only architectures offer reasonable performance, the proposed TriNet-MTL model demonstrates superior generalization capabilities, benefiting from its unified architecture that jointly captures subject-specific, cognitive, and physiological EEG representations.

### Ablation study

To further quantify the contribution of key architectural components within the proposed TriNet-MTL model, we conducted a comprehensive ablation study. The objective of this analysis was to isolate and evaluate the individual impact of the convolutional encoder and the transformer-based temporal encoder on overall model performance across the three classification tasks.

Specifically, we considered the following two ablated variants of the original model:

1. TriNet-MTL without Transformer Encoder: In this configuration, the transformer encoder was removed, and the output from the convolutional encoder was directly forwarded to the task-specific classifiers. This variant tests the importance of long-range temporal modeling via attention mechanisms.
2. TriNet-MTL without Convolutional Encoder: In this variant, the convolutional encoder was omitted, and raw EEG segments were directly processed by the transformer encoder. This assesses the significance of local temporal feature extraction through convolutional operations.

The classification accuracies obtained from these ablated models, alongside the performance of the full TriNet-MTL model, are summarized in Table 3.

**Table 3.** Ablation study results evaluating the contribution of key architectural components

Model variant	Biometric accuracy (%)	Language accuracy (%)	Device accuracy (%)	Average accuracy (%)
Without transformer encoder	88.2	68.4	71.2	75.9
Without convolutional encoder	84.7	65.1	68.9	72.9
Full TriNet-MTL (proposed)	93.9	91.6	92.4	92.6

Performance metrics are reported for model variants excluding the transformer encoder and the convolutional encoder, in comparison with the full TriNet-MTL model. The study underscores the importance of both components in achieving high classification accuracy.

The results clearly demonstrate that both the convolutional encoder and the transformer encoder are critical to achieving optimal performance. The removal of the transformer encoder led to notable performance degradation across all tasks, confirming sits essential role in capturing long-range temporal dependencies and inter-channel correlations within the EEG signals. Similarly, bypassing the convolutional encoder resulted in a further performance drop, indicating that the initial extraction of local temporal features via convolutional operations is fundamental for effective downstream temporal modeling.

The full TriNet-MTL model consistently outperformed its ablated counterparts, highlighting the synergistic benefits of combining convolutional feature extraction with transformer-based temporal modeling. This joint architectural design enables the model to effectively capture both short-term and long-range temporal patterns inherent in auditory-evoked EEG signals, thereby enhancing its discriminative capacity across biometric, cognitive, and physiological classification tasks.

These findings substantiate the architectural design choices of TriNet-MTL and underscore the necessity of both hierarchical temporal convolution and attention-based sequence modeling for robust and generalizable multi-task EEG classification.

## Discussion

This study introduced TriNet-MTL, a unified deep learning framework that jointly addresses biometric identification, auditory stimulus language classification, and auditory delivery modality recognition from auditory-evoked EEG signals. By leveraging both temporal convolution and transformer-based sequence modeling, the model effectively captures a hierarchy of local and global EEG features. Our results demonstrate that TriNet-MTL not only achieves strong classification performance across all tasks but also benefits from multi-task learning through improved generalization and representation sharing. The high accuracy achieved in biometric identification (>93%) validates the potential of auditory-evoked EEG as a viable biometric modality. Compared with traditional EEG-based biometric systems that rely solely on resting-state or motor-evoked potentials, our model shows that task-specific neural responses to auditory stimuli yield rich, individualized patterns useful for identity recognition. These findings align with previous reports showing the effectiveness of auditory evoked potentials (AEPs) in discriminating between individuals based on latency and amplitude features (Picton, 2011; Korkmaz et al., 2020).

In the language classification task (native vs non-native stimuli), TriNet-MTL demonstrated robust performance, highlighting the sensitivity of the auditory system to linguistic familiarity. This supports earlier neurocognitive studies showing differential EEG responses to native and foreign languages (Kotz and Elston-Güttler, 2004). Similarly, the model's ability to classify the auditory delivery modality (in-ear vs bone conduction) with high accuracy (>92%) suggests that physical transmission of sound leaves distinct neurophysiological signatures, a finding also reported by Shoushtarian et al. (2021). Collectively, these results indicate that EEG signals encode not only identity but also context-specific cognitive and sensory information, which can be jointly modeled in a unified framework. An important aspect of TriNet-MTL's design is the integration of multi-task learning (MTL). By training a shared encoder and three task-specific heads, the model learns generalized neural representations that are beneficial across tasks. This joint optimization reduces inter-task interference and encourages task synergy. Compared with baseline models such as single-task transformers and shallow classifiers, TriNet-MTL consistently outperformed in all three classification domains. These results reinforce the value of MTL in scenarios where multiple, related labels can be extracted from the same neural signal (Ruder, 2017). This work builds upon the growing literature of deep learning in EEG decoding. CNN-based approaches like EEGNet (Lawhern et al., 2018) and transformer-based models such as EEG-Former (Wang et al., 2023) have demonstrated success in various EEG applications including motor imagery, attention decoding, and emotion classification. However, few have applied transformers in the context of auditory-evoked EEG, and even fewer have attempted multi-task learning within this domain. Our results extend these previous efforts by demonstrating that transformer encoders can effectively model long-range temporal dependencies in EEG signals, while convolutional layers extract low-level temporal dynamics, a synergy that enhances classification robustness.

Our approach also addresses key limitations of earlier EEG biometric systems that relied heavily on hand-crafted features (Palaniappan and Mandic, 2007) and were often limited to single-task pipelines. By learning end-to-end from raw EEG windows, TriNet-MTL eliminates the need for manual feature engineering and introduces a scalable, data-driven pipeline. Furthermore, our application of sliding window segmentation and dense prediction contributes to improved temporal granularity, a technique known to enhance EEG classification (Roy et al., 2019). One of the major strengths of this work lies in its multifunctional capability. The model is not constrained to a single-purpose task but rather adapts to multiple concurrent objectives without compromising individual task performance. This aligns with real-world requirements in BCI, continuous authentication, and neuroadaptive interfaces, where identity and context must be inferred in real time.

Another strength is the interpretability offered by the model architecture. While transformers are often criticized for being opaque, the modular design of TriNet-MTL, with separate task-specific heads, allows partial transparency in terms of how information is routed for each task. This is a step forward toward explainable AI in EEG systems, though further work is required to enhance interpretability. Despite these strengths, several limitations must be acknowledged. First, the model was evaluated on a single publicly available dataset. While this dataset provides variation in stimulus language and modality, it is limited in terms of demographic diversity, session variability, and recording conditions. Consequently, model generalization to new subjects or sessions remains uncertain. Additionally, although MTL improves performance, it may introduce task imbalance, where one task dominates the loss function unless carefully balanced.

Another limitation is the lack of real-time deployment testing. While the model architecture is computationally efficient, we have not validated latency or throughput under streaming conditions. This is essential for practical applications in BCI and continuous authentication. The results of this study open several promising avenues for future research. First, the issue of cross-session and cross-device generalization remains an important challenge. EEG signals are notoriously nonstationary, and models trained on one session or device often fail to generalize. Addressing this will require exploration of domain adaptation, adversarial training, or contrastive learning techniques to disentangle session-specific artifacts from stable neural features.

Second, enhancing interpretability remains a critical direction. Future iterations of TriNet-MTL could incorporate attention visualization, saliency maps, or layer-wise relevance propagation to identify the spatiotemporal EEG features most relevant for each task. This could also support neuroscientific insights into how identity and cognition are jointly encoded in auditory EEG responses. Third, personalization remains an underexplored dimension. EEG responses vary widely across individuals, and models that support few-shot learning, transfer learning, or meta-learning could improve adaptation to new users with minimal data. This would be particularly useful in practical settings like secure login systems or adaptive cognitive training. Fourth, extending the framework to multimodal systems could further enhance its robustness. For example, integrating EEG with eye-tracking, facial electromyography (EMG), or contextual signals such as environmental noise or user behavior could lead to richer user modeling. These additional modalities can support disambiguation in ambiguous cases and improve real-world accuracy.

Finally, future studies should emphasize larger and more diverse populations. Expanding to different age groups, cultural backgrounds, and cognitive conditions would help evaluate the generalizability and fairness of EEG-based biometric and cognitive inference systems. Moreover, ethical considerations, including data privacy, informed consent, and the potential misuse of biometric neurodata, must be rigorously addressed as the field moves toward practical deployment. In summary, TriNet-MTL represents a robust, flexible, and cognitively informed deep learning architecture for multi-task EEG decoding. By jointly modeling identity, linguistic processing, and sensory modality, the model achieves high performance across all tasks while demonstrating the value of shared neural representation learning. Although limitations remain, this work establishes a foundation for future advances in neural biometrics, cognitive monitoring, and multi-functional brain–computer interfaces. Continued research into generalization, personalization, interpretability, and ethical deployment will be essential to realize the full potential of EEG-based multitask systems in real-world applications.

## Data Availability

The complete source code for TriNet-MTL, including model architecture, training scripts, preprocessing pipeline, and evaluation code, is publicly available. The EEG dataset used in this study is the publicly available Auditory EEG dataset (Accou et al., 2022), accessible at https://physionet.org/content/auditory-eeg/1.0.0/. All preprocessing steps and experimental configurations are documented in the code repository to ensure full reproducibility.

10.1523/ENEURO.0265-25.2025.d1 Data 1 Download Data 1, ZIP file.

## Synthesis

Reviewing Editor: Alexander Soutschek, Ludwig-Maximilians-Universitat Munchen Decisions are customarily a result of the Reviewing Editor and the peer reviewers coming together and discussing their recommendations until a consensus is reached. When revisions are invited, a fact-based synthesis statement explaining their decision and outlining what is needed to prepare a revision will be listed below. The following reviewer(s) agreed to reveal their identity: NONE.

This study investigates the development of a unified deep learning model designed to jointly perform biometric identification, auditory stimulus language classification, and device modality recognition. The approach leverages both physiological and cognitive dimensions of auditory-evoked electroencephalography signals to achieve this integration.

The proposed framework, named Triple-Task Neural Transformer for Multitask Learning, consists of a shared temporal encoder combined with a transformer-based sequence modeling component. Its architecture enables simultaneous learning of task-specific features through three dedicated output modules, each focusing on one of the following objectives: identifying individual users, distinguishing between native and non-native auditory stimuli, and recognizing the delivery mode of the auditory stimulus, either in-ear or bone-conduction. Training is performed with a sliding window method, and optimization relies on a joint cross-entropy loss function applied across all tasks.

Experimental results show that the framework demonstrates robust performance across the three classification objectives. It achieves high accuracy in biometric identification, above ninety-three percent, and shows strong generalization in cognitive state inference. Multitask learning contributes to more effective representation learning by reducing interference between tasks while enhancing synergy among them. Overall, the framework captures both user-specific and cognitively informative patterns from auditory-evoked electroencephalography signals, offering a promising direction for integrated biometric authentication and cognitive state monitoring in real-world applications.

Due to these reasons, the current work will likely inspire further research along similar lines. The following comments should please be taken into account to further improve the manuscript:

1. In particular, the reviewers would like to note recent work at the interface of EEG analysis to capture physiologically meaningful information as well as cognitive states that would further improve the introduction. Namely: Elevated EEG fractal dimension in Parkinson's during timing tasks, Raheleh Davoodi, et al., Chaos 35, 063107 (2025) and Statistically significant features improve binary and multiple motor imagery task predictions from EEGs, Murside Degirmenci, et al., Front. Hum. Neurosci. 11, 1223307 (2023). A good review is also: Complex networks and deep learning for EEG signal analysis, Zhongke Gao, et al., Cogn. Neurodyn. 15, 369-388 (2021). These references will be useful to the authors for a more comprehensive introduction. Currently the introduction is very modest and hardly gives credit to related past research.

2. Also, it would be useful if the authors would make their source code and data available as supplementary material. This would promote the usage of the proposed approach and allow also others to take better advantage of this research, and also allow them to reproduce the results.

3. And finally, it would also improve the paper if the figure captions would be made more self-contained. In addition to what is shown, one could also consider a sentence or two saying what is the main message of each figure, and how to navigate the many details.
