# Supplementary figures and images for: TriNet-MTL: A Multi-Branch Deep Learning Framework for Biometric Identification and Cognitive State Inference from Auditory-Evoked EEG
Source: eNeuro. 2026 Feb 13;13(2):ENEURO.0265-25.2025. doi: 10.1523/ENEURO.0265-25.2025 (PMC12916158; doi:10.1523/ENEURO.0265-25.2025)

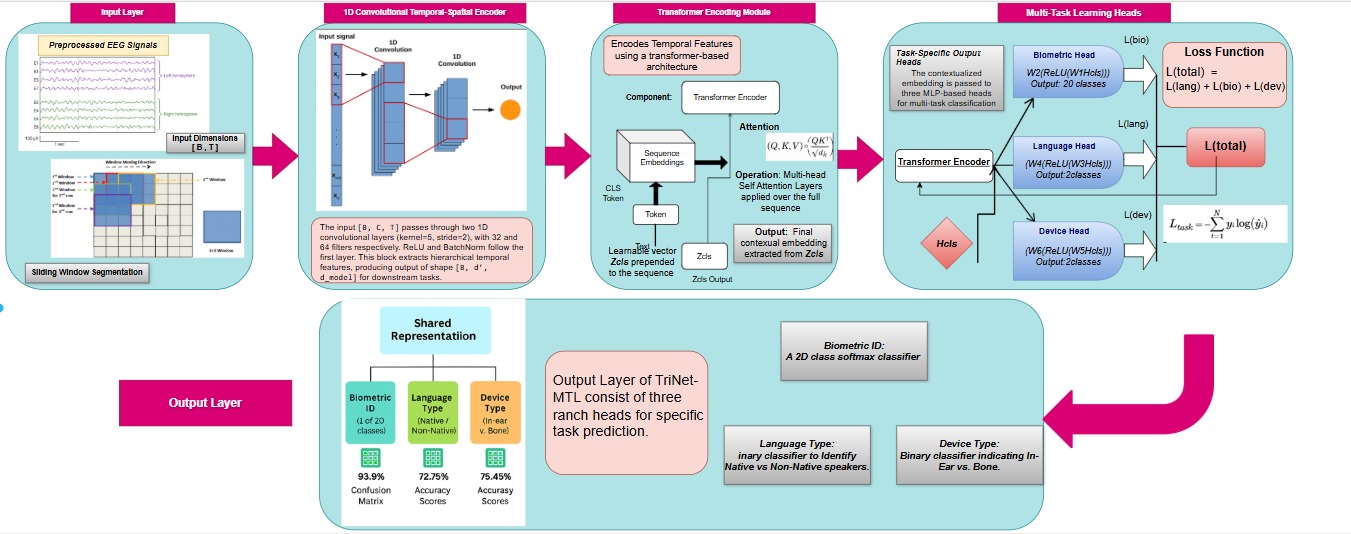

Supplement: Data 1 — Download Data 1, ZIP file. [file eneuro-13-ENEURO.0265-25.2025-s002.zip › TriNet-MTL-main/auditory.jpeg]
